# Hospital admissions among patients with Comorbid Substance Use disorders: a secondary analysis of predictors from the NavSTAR Trial

**DOI:** 10.1186/s13722-024-00463-9

**Published:** 2024-04-28

**Authors:** Courtney D. Nordeck, Sharon M. Kelly, Robert P. Schwartz, Shannon G. Mitchell, Christopher Welsh, Kevin E. O’Grady, Jan Gryczynski

**Affiliations:** 1 Friends Research Institute, 1040 Park Avenue #103, Baltimore, MD USA 21201; 2 Johns Hopkins University School of Medicine, Baltimore, MD USA; 3 University of Maryland School of Medicine, Baltimore, MD USA; 4 University of Maryland, College Park, Baltimore, MD USA

**Keywords:** Opioid use disorder, Substance use disorder, Patient navigation, Healthcare utilization, Hospitalization

## Abstract

**Background:**

Individuals with substance use disorders (SUDs) frequently use acute hospital services. The Navigation Services to Avoid Rehospitalization (NavSTAR) trial found that a patient navigation intervention for hospitalized patients with comorbid SUDs reduced subsequent inpatient admissions compared to treatment-as-usual (TAU).

**Methods:**

This secondary analysis extends previous findings from the NavSTAR trial by examining whether selected patient characteristics independently predicted hospital service utilization and moderated the effect of the NavSTAR intervention. Participants were 400 medical/surgical hospital patients with comorbid SUDs. We analyzed 30- and 90-day inpatient readmissions (one or more readmissions) and cumulative incidence of inpatient admissions through 12 months using multivariable logistic and negative binomial regression, respectively.

**Results:**

Consistent with primary findings and controlling for patient factors, NavSTAR participants were less likely than TAU participants to be readmitted within 30 (*P* = 0.001) and 90 (*P* = 0.03) days and had fewer total readmissions over 12 months (*P* = 0.008). Hospitalization in the previous year (*P* < 0.001) was associated with cumulative readmissions over 12 months, whereas Medicaid insurance (*P =* 0.03) and index diagnoses of infection (*P* = 0.001) and injuries, poisonings, or procedural complications (*P* = 0.004) were associated with fewer readmissions. None of the selected covariates moderated the effect of the NavSTAR intervention.

**Conclusions:**

Previous findings showed that patient navigation could reduce repeat hospital admissions among patients with comorbid SUDs. Several patient factors were independently associated with readmission. Future research should investigate risk factors for hospital readmission among patients with comorbid SUDs to optimize interventions.

**Trial Registration:**

NIH ClinicalTrials.gov NCT02599818, Registered November 9, 2015 https://classic.clinicaltrials.gov/ct2/show/NCT02599818.

**Supplementary Information:**

The online version contains supplementary material available at 10.1186/s13722-024-00463-9.

## Introduction

Substance use disorders (SUDs) and associated health, social, and economic consequences remain a major public health concern in the United States (U.S.). Opioid misuse continues to be a significant national health crisis. In 2020, over 2 million adults in the U.S. met criteria for opioid use disorder (OUD) in the prior year [1], and nearly 70,000 overdose deaths involved opioids, accounting for approximately 75% of all drug overdoses that year [2].

In addition to overdose, substance use is associated with a myriad of physical and mental health conditions directly or indirectly related to its use [3–5]. Consequently, substance use places a high burden on the healthcare system in terms of acute care utilization and related costs [3, 6], as individuals with SUDs are frequent users of hospital services [6–8]. A recent meta-analysis found that, on average, individuals with SUDs visit the emergency department (ED) 4.8 times more often and are admitted as inpatients 7.1 times more than the general population [7]. In particular, the opioid crisis has contributed significantly to skyrocketing rates of hospital utilization, with opioid-related hospitalizations increasing 80% in the 9-year span from 2005 to 2014 [9]. Early research on COVID-19 has shown that individuals with SUDs—especially OUD—were at an increased risk of developing COVID-19 and were more likely to be hospitalized or die compared to individuals with no SUD diagnosis [5].

In response to nationwide upsurges in hospital utilization and costs, the Hospital Readmissions Reduction Program (HRRP) imposes financial penalties for hospitals with high rates of unplanned 30-day readmission for 6 conditions (acute myocardial infarction, chronic obstructive pulmonary disease, heart failure, pneumonia, coronary artery bypass graft surgery, elective primary total hip arthroplasty and/or total knee arthroplasty) [10], whereas Maryland has expanded consideration to all-cause readmissions. Subsequently, there have been major efforts among hospital providers to improve care coordination and develop programs that may effectively reduce potentially avoidable 30-day readmissions. Thus, identifying risk factors associated with acute hospital utilization may be beneficial in guiding the development of such interventions.

Although studies have generally focused on readmission among patients with health conditions included in HRRP, some behavioral health studies have evaluated risk factors for readmission in patients with SUDs. Demographic variables such as male sex [11–16] and older age [11, 14–16] have been found to be associated with 30-day readmission, as have health-related factors such as severity of illness [15, 16], presence of chronic physical conditions [11–16], and co-morbid SUDs and mental health disorders [12–14]. Other factors associated with 30-day rehospitalization include enrollment in Medicaid and/or Medicare [11, 13–16], longer stay at index admission [12, 17], discharge against medical advice [13, 15], and a recent inpatient hospitalization (prior to index hospitalization) [12]. Some behavioral health studies have used 90 days as a benchmark for readmission and have reported similar findings [17–22], with most studies showing a significant relationship between co-occurring psychiatric conditions and SUDs and greater likelihood of 90-day readmission [18–22].

The parent study for the current analysis is a randomized clinical trial of a patient navigation intervention called Navigation Services to Avoid Rehospitalization (NavSTAR) conducted among 400 hospitalized patients with comorbid SUDs recruited from a large academic hospital in Baltimore City [23, 24]. The NavSTAR intervention consisted of three months of proactive, individualized case management, service linkage, and motivational support to reduce acute healthcare utilization and facilitate community-based SUD treatment following hospital discharge. The study examined the effectiveness of the NavSTAR intervention in reducing readmissions compared to treatment-as-usual (TAU). Study findings showed that, on an intention-to-treat basis, NavSTAR participants had significantly lower subsequent hospital service utilization (the primary outcome) compared to TAU, including a strong effect on 30-day inpatient readmissions (15.5% NavSTAR vs. 30.0% TAU) [23]. In the current secondary study, we extend the primary analysis to examine the role of selected patient characteristics in (a) independently predicting hospital readmissions, and (b) moderating the effect of the NavSTAR intervention on hospital readmissions. The goal of these analyses was to gain a better understanding of which patient characteristics are associated with hospital readmissions in this sample and to explore how certain patient groups may be more or less likely to benefit from the intervention. The examination of predictors and potential moderators is important in considering whether there were subgroups within the study sample for which the intervention was more or less beneficial, which could improve implementation of such an intervention in other settings and guide future research.

## Methods

### Study setting and Sample

Participants were 400 medical/surgical patients with co-occurring SUDs recruited from the University of Maryland Medical Center (UMMC) and who were receiving services from the hospital-based SUD consultation service at the time of enrollment. The NavSTAR intervention was an add-on service for patients that was initiated in the hospital by the study team prior to discharge and continued for three months post-discharge. Study inclusion criteria were: (1) ≥ 18 years of age; (2) meet DSM-5 criteria for opioid, cocaine, and/or alcohol use disorder; and (3) willingness and ability to provide informed consent in English. Individuals were excluded if they were: (1) enrolled in SUD treatment 30 days prior to hospitalization; (2) living outside of Baltimore City; (3) pregnant; (4) expected to be discharged to a long-term or terminal inpatient facility (e.g., hospice); or (5) hospitalized for a suicide attempt. The study was approved by Friends Research Institute’s and University of Maryland School of Medicine’s Institutional Review Boards.

Hospitalized patients who were deemed medically stable by the SUD consultation service and showed interest in study participation were referred to study staff between March 2016 and May 2018. A Research Assistant (RA) screened individuals for preliminary eligibility, obtained informed consent, and conducted a baseline interview. Individuals deemed eligible were then assigned to study condition (1:1 basis) in blocks of 2, 4, or 6. Detailed information on study methods have been described previously [23, 24].

### Study Condition

#### Treatment as Usual (TAU)

Participants assigned to TAU received usual care from the hospital’s medical team and SUD consultation service team, who provided counseling, withdrawal management, initiation of methadone or buprenorphine (if appropriate), and referrals to community-based treatment. However, the role of the consultation service was limited to the acute episode, lacking capacity to follow patient progress post-discharge.

#### Navigation Services to Avoid Rehospitalization (NavSTAR)

The NavSTAR intervention was designed by the study team to serve as an extension of the hospital’s SUD consultation service and utilized patient navigation to promote engagement in post-discharge care using a combination of motivational interviewing techniques and proactive case management/care coordination services [24]. Participants in the NavSTAR condition received the same services as TAU participants, plus three months of patient navigation services post-discharge. The patient navigators (Masters-level licensed social workers) met with participants at bedside for a preliminary session to establish rapport, assess medical and substance use treatment needs, deliver motivational counseling, and develop a post-discharge plan. Upon discharge, navigators worked with patients for up to three months to provide support, link participants to resources for obtaining basic needs (e.g., housing, transportation), and address barriers to engaging in care and maintaining health.

### Outcome variables

Study outcome measures for the current analysis were: (1) any (i.e., ≥ 1) inpatient readmissions within 30 days or (2) within 90 days of discharge from the index hospitalization (yes/no); and (3) cumulative number of readmissions with 12 months of discharge from the index hospitalization. Data on inpatient admissions were obtained from the Chesapeake Regional Information System for our Patients (CRISP) health information exchange that links patients’ electronic health records from all hospitals in the Maryland and Washington, DC region (except Veterans Affairs) [25].

### Explanatory variables

In addition to study condition (NavSTAR vs. TAU), explanatory variables included in the statistical model were the baseline demographic variables of gender, race (white vs. non-white), and age, obtained from the Addiction Severity Index (ASI)-Lite [26] administered at baseline. Variables indicating whether participants met criteria for DSM-5 OUD, cocaine use disorder (CUD), and/or alcohol use disorder (AUD) in the 30 days prior to index hospitalization were included and were calculated using responses to items from a modified World Mental Health Composite International Diagnostic Interview that map to DSM-5 SUD diagnostic criteria [27]. Additional explanatory variables were: hospital admission in the year prior to index hospitalization (yes/no) and incarceration in the year prior to index hospitalization (yes/no) as self-reported on a modified Economic Form-90 [28]; injection drug use in the prior three months of hospitalization (yes/no) as self-reported on the Risk Assessment Battery [29]; having Medicaid as primary insurance (vs. Medicare/uninsured/private/other insurance), current experiences of homelessness (yes/no), documentation of a mental health diagnosis (yes/no), and length of stay, which were abstracted from the research hospital’s electronic health record (EHR). Reason for the index hospitalization was categorized using the Medical Dictionary for Regulatory Activities (MedDRA) and were collapsed into the following categories based on prevalence: infections; injuries, poisoning, and procedural complications; cardiac disorders; gastrointestinal disorders; and other conditions [30].

### Statistical analysis

Analyses were conducted on an intention-to-treat basis using Stata version 16. In the primary outcomes paper, we reported on hospital readmission for NavSTAR and TAU arms but did so without considering the role of other predictor variables or potential moderators. Thus, the current study extends the prior analysis by considering other explanatory variables. Logistic regression was utilized to examine variables that predicted the two binary outcomes of any inpatient readmission within 30 days and 90 days, respectively. Negative binomial regression was used to examine cumulative inpatient readmissions over 12 months. Predictor variables in the models included study arm and the explanatory variables (the aforementioned patient-level variables). Because there was insufficient information available to posit firm directional hypotheses about potential moderators of the effectiveness of the Patient Navigation intervention, we examined the possible role of the explanatory variables as moderators of the intervention effect on an exploratory basis. These putative moderating factors were examined from the perspective of heterogeneity of treatment effects variables specified via multiplicative interactions as part of the statistical model a posteriori [31]. This approach sought to determine the extent to which the relative effectiveness of the NavSTAR and TAU arms differed based on these participant characteristics. Moderator variables were considered one at a time and dropped if not significant at the 0.05 level. The final models included all explanatory variables plus any interactions that remained statistically significant (if any) in the analysis of a given outcome. To examine the nature of the interaction effect, model-based predictions and contrasts were computed to compare NavSTAR and TAU at different values of the moderator variable.

## Results

### Participants

The sample of 400 participants (200 NavSTAR, 200 TAU) consisted of 228 men (57.0%); mean (SD) age was 45.1 (12.3) years (Table 1). The majority of the sample was Black (*n* = 222, 55.5%), had Medicaid as their primary source of insurance (86.0%), and reported at least one hospitalization in the year prior to enrollment (65.5%). Over three-quarters (78.5%) of the sample met criteria for DSM-5 OUD, just over half (53.5%) met criteria for CUD, and more than a third (35.3%) met criteria for AUD.

**Table 1:** Participant characteristics by hospital readmission within 30, 90, and 365 days of discharge (*N* = 400)

	Total Sample	Readmitted (once or more) within:		
	(*N =* 400)	30 Days (*n* = 91)	90 Days (*n* = 167)	365 Days (*n* = 277)
Study condition, *n* (%)				
NavSTAR	200 (50.0)	31 (15.5)	73 (36.5)	131 (65.5)
TAU	200 (50.0)	60 (30.0)	94 (47.0)	146 (73.0)
Gender, *n* (%)				
Male	228 (57.0)	59 (25.9)	96 (42.1)	154 (67.5)
Female	172 (43.0)	32 (18.6)	71 (41.3)	123 (71.5)
Race, *n* (%)				
White	169 (42.3)	39 (23.1)	75 (44.4)	119 (70.4)
Non-White^*^	231 (57.8)	52 (22.5)	92 (39.8)	158 (68.4)
Age				
Mean (*SD*)	45.1 (12.3)	44.9 (12.5)	44.9 (12.4)	45.5 (12.2)
Homelessness, *n* (%)	172 (43.0)	45 (49.5)	80 (47.9)	126 (45.5)
Insurance, *n* (%)				
Medicaid	344 (86.0)	74 (21.5)	141 (41.0)	231 (67.2)
Medicare/private/uninsured/other	56 (14.0)	17 (30.4)	26 (46.4)	46 (82.1)
Length of stay at index hospitalization				
Mean days, (*SD*)	6.9 (5.6)	7.9 (6.7)	7.0 (5.7)	7.1 (5.7)
Meets DSM-5 AUD criteria, *n* (%)	141 (35.3)	34 (24.1)	61 (43.3)	103 (73.1)
Meets DSM-5 OUD criteria, *n* (%)	314 (78.5)	71 (22.6)	130 (41.4)	214 (68.2)
Meets DSM-5 CUD criteria, *n* (%)	214 (53.5)	46 (21.5)	90 (42.1)	149 (69.6)
Injection drug use, past 3 months, n (%)				
Any mental health disorder, *n* (%)	123 (30.8)	30 (33.0)	60 (35.9)	97 (35.0)
Hospitalization, past year, *n* (%)	262 (65.5)	64 (24.4)	122 (46.6)	188 (71.8)
Incarceration, past year, *n* (%)	83 (20.8)	16 (17.6)	32 (19.2)	55 (19.9)
Index diagnosis				
Infections	192 (48.0)	39 (42.9)	75 (44.9)	126 (45.5)
Injuries and poisonings	43 (10.8)	14 (15.4)	16 (9.6)	26 (9.4)
Cardiac disorders	32 (8.0)	5 (5.5)	16 (9.6)	27 (9.8)
Gastrointestinal disorders	28 (7.0)	7 (7.7)	11 (6.6)	22 (7.9)
Other	105 (26.2)	26 (28.6)	49 (29.3)	76 (27.4)

*NotesNavSTAR =* Navigation Services to Avoid Rehospitalization; *TAU* = treatment as usual; *ASI* = Addiction Severity Index; *DSM-5 =* Diagnostic Statistical Manual, 5th Edition; *AUD* = alcohol use disorder; *OUD* = opioid use disorder; *CUD* = cocaine use disorder

Column percentages for the Total Sample may not sum to 100% due to rounding

* Includes participants who identified as Black (*n* = 222), Hispanic (*n* = 6), Asian (*n* = 2), and Native American (*n* = 1)

According to discharge diagnoses obtained from participants’ EHRs, participants were hospitalized for a variety of medical reasons, primarily infections (48%), injuries/poisonings (10.8%), cardiac disorders (8%), and gastrointestinal disorders (7%). The prevalence of diagnostic categories using MedDRA coding is presented in Supplemental Table 1.

### Any inpatient readmission within 30 days

Ninety-one participants (22.8%) had a 30-day inpatient readmission. Table 2 shows the results of the logistic regression analysis. NavSTAR participants were significantly less likely to experience 30-day inpatient readmission compared to TAU participants (Adjusted Odds Ratio [AOR] = 0.42, 95% Confidence Interval [95% CI] = 0.25, 0.70, *P =* 0.001). The remaining explanatory variables failed to reach significance. There were no significant interactions with intervention condition in moderation testing.

**Table 2:** Predictors of any inpatient hospital readmission within 30 and 90 days of discharge (*N* = 400)

	Any 30-Day Inpatient Readmission				Any 90-Day Inpatient Readmission		
	*AOR*	*95% CI*	*P*		*AOR*	*95% CI*	*P*
NavSTAR (reference: TAU)	0.42	0.25, 0.70	0.001		0.62	0.41, 0.94	0.03
Female (reference: Male)	0.64	0.37, 1.12	0.12		0.95	0.60, 1.51	0.84
White (reference: non-White^*^)	1.06	0.56, 2.01	0.87		1.35	0.78, 2.33	0.29
Age	0.98	0.96, 1.01	0.17		0.99	0.97, 1.01	0.25
Homelessness (reference: stably housed)	1.39	0.79, 2.45	0.25		1.35	0.84, 2.16	0.22
Medicaid insurance (reference: other^**^)	0.61	0.30, 1.26	0.18		0.77	0.41, 1.46	0.43
Length of stay at index hospitalization	1.04	1.00, 1.09	0.06		1.00	0.97, 1.04	0.82
Meets DSM-5 AUD criteria (reference: no)	1.08	0.55, 2.11	0.83		1.01	0.58, 1.77	0.97
Meets DSM-5 OUD criteria (reference: no)	1.22	0.56, 2.69	0.61		1.14	0.59, 2.19	0.70
Meets DSM-5 CUD criteria (reference: no)	0.96	0.56, 1.66	0.89		1.07	0.68, 1.69	0.77
Injection drug use (reference: no IDU)	0.94	0.44, 2.01	0.88		0.80	0.42, 1.51	0.49
Comorbid mental health disorder	1.10	0.64, 1.91	0.73		1.43	0.90, 2.27	0.13
Hospitalization, past year	1.30	0.76, 2.23	0.34		1.75	1.12, 2.74	0.01
Incarceration, past year	0.69	0.36, 1.35	0.28		0.77	0.45, 1.34	0.36
Index diagnosis (reference: other)							
Infections	0.67	0.32, 1.38	0.28		0.63	0.34, 1.15	0.14
Injuries and poisonings	1.25	0.54, 2.87	0.61		0.64	0.30, 1.38	0.25
Cardiac disorders	0.55	0.18, 1.64	0.28		1.26	0.55, 2.88	0.58
Gastrointestinal disorders	0.96	0.34, 2.68	0.94		0.64	0.26, 1.58	0.33

*Notes* AOR  = Adjusted Odds Ratio; *CI* = Confidence Interval; *NavSTAR* = Navigation Services to Avoid Rehospitalization; *TAU* = Treatment as Usual; *DSM-5* = Diagnostic Statistical Manual, Fifth Edition; *AUD* = alcohol use disorder; *OUD* = opioid use disorder; *CUD* = cocaine use disorder

* Includes participants who identified as Black, Hispanic, Asian, and/or Native American

** Includes Medicare, private insurance, Veterans Administration insurance, no insurance, and other insurance

### Any inpatient readmission within 90 days

Over two-fifths of participants (*n* = 167, 41.8%) had a 90-day inpatient readmission. NavSTAR participants remained less likely than TAU participants to be readmitted within 90 days (AOR = 0.62, 95% CI = 0.41, 0.94, *P* = 0.03). Participants who reported a prior-year hospitalization were more likely to be readmitted within 90 days than participants without a prior-year hospitalization (AOR = 1.75, 95% CI = 1.12, 2.74, *P* = 0.01). None of the interaction effects were significant, and thus were dropped from the model.

### Incidence of inpatient readmissions over 12 months

In a multivariable negative binomial regression (Table 3), hospitalization in the prior year (Incidence Rate Ratio [IRR] = 1.80, 95% CI = 1.38, 2.34, *P* < 0.001) was associated with more inpatient admissions over 12 months of follow-up, while Medicaid insurance coverage (IRR = 0.66, 95% CI = 0.46, 0.96, *P* = 0.03), index diagnoses related to infection (IRR = 0.57, 95% CI = 0.40, 0.79, *P* = 0.001), and index diagnoses related injury, poisoning, or procedural complications (IRR = 0.52, 95% CI = 0.34, 0.81, *P* = 0.004) were associated with fewer inpatient admissions. Finally, the NavSTAR intervention was associated with fewer inpatient admissions over 12-months of follow-up (IRR = 0.72, 95% CI = 0.57, 0.92, *P* = 0.008). The remaining explanatory variables failed to reach significance. None of the interaction effects were significant and were dropped from the model.

**Table 3:** Predictors of cumulative inpatient readmissions within 12 months of discharge (*N =* 400)

	Cumulative Inpatient Readmissions Over 12 months		
	*IRR*	*95% CI*	*P*
NavSTAR (reference: TAU)	0.72	0.57, 0.92	0.008
Female (reference: Male)	0.96	0.73, 1.25	0.74
White (reference: non-White^*^)	1.05	0.78, 1.43	0.74
Age	0.99	0.98, 1.01	0.28
Homelessness (reference: stably housed)	1.20	0.91, 1.60	0.20
Medicaid insurance (reference: other^**^)	0.66	0.46, 0.96	0.03
Length of stay at index hospitalization	1.01	0.99, 1.03	0.46
Meets DSM-5 AUD criteria (reference: no)	0.90	0.64, 1.27	0.55
Meets DSM-5 OUD criteria (reference: no)	1.01	0.69, 1.48	0.95
Meets DSM-5 CUD criteria (reference: no)	1.18	0.90, 1.55	0.22
Injection drug use (reference: no IDU)	0.87	0.60, 1.26	0.46
Comorbid mental health disorder	1.20	0.92, 1.57	0.18
Hospitalization, past year	1.80	1.38, 2.34	< 0.001
Incarceration, past year	0.81	0.59, 1.11	0.19
Index diagnosis (reference: other)			
Infections	0.57	0.40, 0.79	0.001
Injuries and poisonings	0.52	0.34, 0.81	0.004
Cardiac disorders	1.00	0.62, 1.60	1.00
Gastrointestinal disorders	0.59	0.34, 1.01	0.06

*Notes* IRR  = Incidence Rate Ratio; *CI* = Confidence Interval; *NavSTAR* = Navigation Services to Avoid Rehospitalization; *TAU* = Treatment as Usual; *DSM-5* = Diagnostic Statistical Manual, Fifth Edition; *AUD* = alcohol use disorder; *OUD* = opioid use disorder; *CUD* = cocaine use disorder

* Includes participants who identified as Black, Hispanic, Asian, and/or Native American

** Includes Medicare, private insurance, Veterans Administration insurance, no insurance, and other insurance

## Discussion

The findings in the current study corroborate findings from the NavSTAR trial showing the effectiveness of a patient navigation service in reducing inpatient readmission among patients with comorbid SUD [23], with the intervention effect remaining intact when controlling for various participant characteristics and potential confounds. Moreover, we examined the role of a set of explanatory variables as predictors or potential moderators of intervention effects associated with hospital readmissions [23, 24]. While there were no significant moderators identified in models predicting any readmission within 30 or 90 days or cumulative readmissions across 12-months follow-up, some caution is warranted based on the exploratory nature of the moderation analysis.

This study identified several participant characteristics that were independently associated with hospital service utilization, controlling for the possible impact of the intervention. We found that patients who reported a hospitalization within the year prior to enrollment were significantly more likely to be readmitted within 90-days and to have greater incidence of cumulative admissions across 12-months of follow-up. This is consistent with research that has found higher risk of readmission among patients with prior inpatient stays among populations with certain conditions such as mental or substance use disorder [12] and COPD [32].

In the analysis of cumulative readmissions, we also found that the incidence of readmissions differed based on diagnosis at the index hospitalization. Patients initially hospitalized for an infection or for injuries, poisonings, and procedural complications had fewer readmissions over the course of 12 months compared to patients initially hospitalized for other conditions. Still, infections were the most common reason for which participants were initially hospitalized. Moreover, nearly half of those who were initially hospitalized for infection in this sample had at least one subsequent hospitalization within 12-months. In a nationally representative study of all discharges across US acute care hospitals, researchers found that, among individuals hospitalized for serious infections, OUD was associated with longer length of stay and higher odds of discharge to post-acute care facilities or patient-directed discharges, highlighting significant disparities in post-discharge options for those with OUD [33]. While there is extensive literature related to high rates of hospitalization from drug-related infections [34], studies have found that adequate infectious disease intervention during hospitalization may reduce the risk of subsequent readmissions [35] and that receipt of MOUD is associated with lower one-year readmissions among those hospitalized with skin and soft tissue infections [36, 37]. Furthermore, research has demonstrated an undermanagement of comorbid substance use among hospitalized patients with drug-related infections [38] that could be improved through the coordinated efforts of specialized hospital-based addiction care and attending medical teams. Given the harms of the unregulated drug supply, particularly with the recent increase in xylazine that contributes to distinct skin wounds, patient-centered management of both substance use and infections are urgently needed in medical settings. Index hospitalization related to injuries, poisonings, and procedural complications could be reflective of acute conditions that are not associated with risk of subsequent hospitalizations compared to chronic conditions such as COPD and heart failure.

Finally, we found lower incidence of cumulative inpatient admissions among participants with Medicaid insurance coverage, which is generally contrary to other studies. It is important to note that a large majority of the sample was covered by Medicaid (86%), which may impact the ability to detect differences among the other insurance types.

This study has several limitations. Participants were recruited from a single urban university hospital in the Mid-Atlantic region; therefore, generalizability is limited. Relative to studies using larger secondary data sets [11, 13, 14, 16], our analyses were limited by our sample size and we did not have measures of some of the factors previously found to be associated with rehospitalization in the literature, such as other comorbidities at the index hospitalization. Thus, it is possible that some important correlates of hospital readmission were not examined. The relatively small sample size may have left us underpowered to detect differences, particularly for the analysis of moderating effects. We also examined hospitalization outcomes only, but there are other important patient-centered outcomes, such as SUD treatment entry, that were a targeted goal of the patient navigation intervention and merit investigation. Further, recruitment in this study primarily took place in the hospital’s internal medicine, trauma, and surgery units. Recruitment from other patient populations (e.g., labor and delivery, inpatient psychiatry) could have led to different results.

## Conclusion

NavSTAR is a promising intervention shown to be effective in reducing inpatient hospital readmission. The current analysis corroborated findings supporting the NavSTAR intervention in reducing inpatient readmission, even when controlling for various patient-level factors. Importantly, we identified some patient characteristics that were independently associated with subsequent hospital readmissions. Hospital service utilization presents an opportunity to engage vulnerable patients and administer interventions to facilitate SUD treatment entry and disrupt or slow the cycle of repeat hospital visits.

### Electronic supplementary material

Below is the link to the electronic supplementary material.

Supplementary Material 1

## Data Availability

The deidentified data sets can be shared pending approval of a research proposal and a written data sharing and use agreement with Friends Research Institute. For inquiries, please contact Dr. Gryczynski (e-mail, jgryczynski@friendsresearch.org).
