# Supplementary material for: Hospital admissions among patients with Comorbid Substance Use disorders: a secondary analysis of predictors from the NavSTAR Trial
Source: Addict Sci Clin Pract. 2024 Apr 28;19:33. doi: 10.1186/s13722-024-00463-9 (PMC11056040; doi:10.1186/s13722-024-00463-9)
Supplement: Supplementary file 1 — Supplementary Material 1 [file 13722_2024_463_MOESM1_ESM.docx]

**SUPPLEMENTAL TABLE 1.** Categories of index diagnosis using the Medical Dictionary of Regulatory Activity.

|  | Total (n=400) |
| --- | --- |
| Blood disorders | 1 (0.3) |
| Cardiac disorders | 32 (8.0) |
| Congenital, familial, genetic disorders | 2 (0.5) |
| Endocrine disorders | 1 (0.3) |
| Gastrointestinal disorders | 28 (7.0) |
| General disorders/administration site conditions | 15 (3.8) |
| Hepatobiliary disorders | 9 (2.3) |
| Immune system disorders | 3 (0.8) |
| Infections and infestations | 192 (48.0) |
| Metabolism and nutrition | 8 (2.0) |
| Musculoskeletal/connective tissue disorders | 5 (1.3) |
| Neoplasms (benign, malignant, unspecified) | 2 (0.5) |
| Nervous system disorders | 9 (2.3) |
| Product issues | 1 (0.3) |
| Psychiatric disorders | 3 (0.8) |
| Renal and urinary disorders | 6 (1.5) |
| Respiratory, thoracic, medistatinal disorders | 23 (5.8) |
| Vascular disorders | 17 (4.3) |
| Injury, poisoning, procedural complications | 43 (10.8) |
